# Data on self-esteem among adolescents in India

**DOI:** 10.6026/973206300191086

**Published:** 2023-11-30

**Authors:** Mahalakshmi B, Sivasubramanian N, Urviben Yogeshkumar Patel, Ekambaram Gnanadesigan

**Affiliations:** 1 Department of Paediatric Nursing, Nootan College of Nursing, Sankalchand Patel University, Visnagar, Gujarat - 384315, India; 2 Department of Psychiatric Nursing, Nootan College of Nursing, Sankalchand Patel University, Visnagar, Gujarat - 384315, India; 3 Department of Physiology, Nootan Medical College and Research Centre, Sankalchand Patel University,Visnagar, Gujarat - 384315, India

**Keywords:** Effect, assertiveness training, self-esteem, adolescents.

## Abstract

A person's total perception of his or her value or worth is referred to as self-esteem. It may serve as a proxy for how much a person "values, approves of, appreciates, prizes, or likes [him or herself]". The study's major goals were to assess adolescents' levels of self-esteem and examine the impact assertiveness training had on those adolescents' self-esteem. The research design selected for the study was pre-experimental one group pre-test and post-test research design". Anon-probability convenience sampling technique was used to obtain a sample of 60 adolescents who fulfilled the inclusion criteria. Rosenberg Self-Esteem Scale was the study's primary instrument, a 10-item questionnaire that a person fills out and scores on a 0-3 scale, containing both positive and negative items, is used to measure one's degree of self-esteem. In this case, questions 2, 5, 6, 8, and 9 had lower scores than questions 1,3,4,7 and 10. The Likert scale looks like this: Strongly disagree, strongly agree, agree, and disagree. The mean Self-Esteem score prior to the test was 11.33 with a standard deviation of 1.28, whereas the mean Self-Esteem score after the test was 21.16 with a standard deviation of 1.94. The mean difference of 9.83 is significant at 0.001 levels. The 't' value of 33.4 was higher than the table value. This study provides evidence of adolescents' self-esteem has been improved through assertiveness training.

## Background:

Self-esteem is quite simply one's attitude towards oneself (1965). He described it as a "favorable or unfavorable attitude towards the self". -Morris Rosenberg. The amount of value people place on them is the fundamental meaning of self-esteem. It serves as the evaluative component of self-knowledge. Early on, William James (1890) suggested that self-esteem was a result that depended on how one's successes compared to one's pretensions, as expressed in the following equation: self-esteem=successes pretensions [1]. Self-esteem is a personal evaluation of one's entire value [2]. Every person is capable of evaluating and experiencing it. It is a comprehensive assessment of each person's value. A good sense of worth affects our drive, values, and healthy way of living. When it comes to adolescents, self-esteem varies between the early and late adolescent years. This would suggest that self-esteem has enduring effect on adolescent period [3]. Self-esteem is also dimensional, associates with significant life outcomes, such as physiological, psychological health, academic success, and personality traits. Teenagers with low self-worth may often struggle with depression or eating disorders. Therefore, it is crucial to examine and raise adolescents' self-esteem. A form of therapy called assertive training teaches patients confidence-boosting practices [4]. It aids people in learning to articulate their needs and wants more effectively, particularly those who have a tendency to be passive in doing so. According to a study, self-esteem and academic success are two aspects that assertiveness does not significantly influence. The researcher also discovered that 13%-15% of adolescents in India exhibited passive behaviour [5]. In The location of Mehsana, 25% to 27% of adolescents had been influenced by low levels of assertive behaviour, and 47% to 49 percent of adolescents in Gujarat exhibited low levels of this behaviour [6]. Therefore, it is of interest to assess the assertiveness training on self-esteem among adolescents at Visnagar, Mehsana, Gujarat, India.

## Material and Methods:

## Research Approach:

In this present study, descriptive research approach was adopted [7]

## Research design:

This study employed one group pre-test and post-test design.

## Target population:

60 adolescents from Higher Secondary School and colleges at Visnagar, who met the criteria, were the target population in the current research [8,9].

## Sampling Technique:

Anon-probability convenient sampling method was chosen for the current research [10].

## Sample size and sampling criteria:

The sample consists of 60 adolescents studying at Nootan Sarva Vidhyalaya Higher Secondary School, Visnagar. Adolescents who were studying at Nootan Sarva Vidyalaya Higher Secondary School and university, Visnagar between the ages of 13 to 19 and who were able to communicate in Gujarati, Hindi, or English were included. Adolescents who were unwilling to participate and Adolescents who were not available at the time of data collection were excluded from the study.

## Study description:

Demographic variables such as Age, religion, level of education, academic performance, family type, birth order, monthly family income, residential location, co-curricular activity, father's and mother's educational backgrounds, father's and mother's occupations, and age are among the demographic variables were included in this study. The Rosenberg Self-Esteem Scale was the study's primary instrument. A questionnaire method was employed for the investigation. There were two parts to the Tool. The Rosenberg Self-Esteem range is a 10-item questionnaire comprising positive and negative items that are meant to gauge an individual's level of self-esteem (Table 1). Each response is scored on a range of 0 to 3. In this case, questions 2,5,6,8 and 9 had lower scores than questions 1,3,4,7 and 10. The Likert scale looks like this: Strongly disagree, strongly agree, agree, and disagree. The present study was approved by the Institutional ethics committee and an informed consent was taken from all the subjects after explaining the test procedures and the goal of the study in local language.

## Data collection method:

The quality and accuracy of the data obtained are determined by the data collection procedures, which are essential to the research process [11,12]. To conduct the study formal approval was acquired from the Principal and Head of the Department at Nootan College of Nursing, the Head Master, and the Chief Educational Officer at Nootan SarvaVidhyalaya Higher Secondary School. Before the study was done, a brief introduction and explanation of the nature and purpose of the study's intervention were explained. All subjects' parents provided both written and verbal consent. Each week, 10 subjects were chosen. On the first day of the pre-test, the Rosenberg Self-Esteem Scale was used to gauge the teenagers' level of self-esteem. Training in assertiveness was provided, and it had eight components: circumstance, respect for others, and appreciation of oneself, appreciation of others, mirror acting, mirror talking, self-improvement exercises, and narrative.

## Statistical Analysis:

Data analysis techniques include descriptive and inferential statistics [13]. Data are conveyed as mean ± standard deviation (SD). The acquired data were analyzed using MANCOVA and student t-test in SPSS (version 16) at the significant level of P - value 0.05.

## Results:

According to given data, 42 adolescents (or 70%) had poor levels of self-esteem, whereas 18 adolescents (or 30%) had moderate levels. The majority of the teenagers (38/63.3%) who participated in the post-intervention assertiveness training assessment had high levels of self-esteem, while 22 (36.7%) had average level.

**Figure 1:**
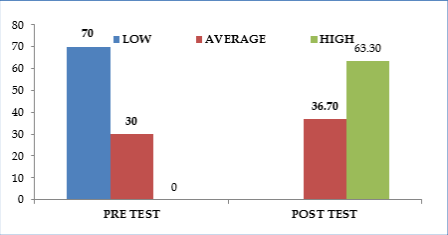
The cylinder diagram quotes the distribution of adolescents according to their level of Pre and post-test level of Self-Esteem.

According to Figure 1 42 (70%) of the adolescents who completed the pre-test had low levels of self-esteem, whereas 18 (30%) had average levels Table 2. After receiving assertiveness training, the majority of the adolescents 38 out of them had high levels of self-esteem in the post-test, while 22 out of them or 36.7% had average levels.

The above table depicts the Mean of the Pre-test and Post-test was11.33 and21.16 respectively and the Standard Deviation of the Pre-test and Post-test was 1.2 and 1.9 respectively. The Mean difference was 9.83. The paired 't' - test value was 33.4.This showed that there was a significant difference between the pre-test and post-test levels of Self-Esteem among adolescents.

**Figure 2:**
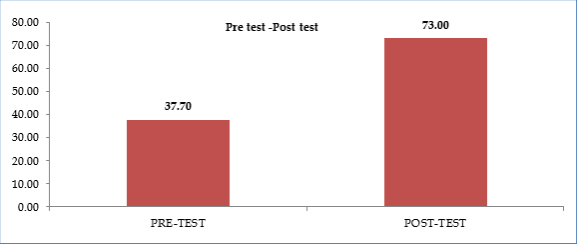
The cylinder diagram depicts the effectiveness of assertiveness training on self-esteem among adolescents.

Figure 2 depicts the Mean of the Pre-test and Post-test was 11.33 and 21.16 respectively and the Standard Deviation of the Pre-test and Post-test was 1.2 and 1.9 respectively. The Mean difference was 9.83. The paired t - 'test value was 33.4. This showed that there was a significant difference between the pre-test and post-test levels of Self-Esteem among adolescents Table 3.

Table 4 explains the association between the levels of Self-Esteem among adolescents with their selected socio-demographic variables. Chi-square analysis revealed that there was an association between the post-test level of Self-Esteem and age(13 years), monthly income(belowRs.3000,)and mothers' educational status (No formal education). All other variables were not significantly associated among the adolescents with their post-test scores.

## Discussion:

The purpose of this research was to find out how assertiveness training improved the self-esteem of adolescents who participated in Visnagar School. On the first day, a pre-test was done, and the adolescents' level of self-esteem was assessed by the Rosenberg Self-Esteem Scale. From the day of the pre-test until the day of the post-test, assertiveness training with eight components - situation, respecting others, self-appreciation, appreciation of others, mirror talking, mirror acting exercise, self-improvement exercise, and storytelling was delivered. The mean post-test self-esteem score was 21.16, whereas the pre-test self-esteem score was 11.33; this variation in mean resulted from the assertiveness training interventions and was not by chance. This demonstrated that there was a substantial difference in the adolescents' self-esteem levels before and after taking the test. The results of the chi-square analysis indicated that it was related to the level of self-esteem at the post-test. The current study has been confirmed by Khansa Malik and Bince Varghese [10], who conducted a study on the impact of assertiveness training on adolescents' self-esteem in 2020. Our results were also in agreement with Parry (2017) [5]. An Indian Outlook found that the training had a positive impact on adolescents' self-esteem. The majority of the outcomes were in the pre-test. Following the test, the majority of subjects (68.33%) had excellent self-esteem, followed by 31.66% who had few problems with self-esteem and no one who fell into the categories of low and depressed self-esteem (61.66% had low self-esteem, 38.33% had no problems, and 31.66% experienced but not all problems) [10]. The present study findings were consistent [14], their results displayed that the assertiveness training on self-esteem and assertive behaviour among adolescents was rational and cost effective tactic. Another study [15,16] found that assertiveness training was successful (p 0.05) in raising self-esteem levels and enhancing assertive conduct in adolescent girls, and the results also show that it becomes better with time.

## Conclusion:

Data shows that training in assertiveness was successful in raising adolescents' self-esteem. This is affordable and works well to boost self-esteem.

## Figures and Tables

**Table 1:** Scoring and Interpretation

**Score**	**Level of self-esteem**
Low	0 -14
Average	15-25
High Self-Esteem	26-30

**Table 2:** Frequency and percentage distribution of adolescents according to the level of self-esteem

**Level of self esteem**	**Pre-test**		**Post-test**	
	**F**	**%**	**F**	**%**
Low	42	70.00%	0	0.00%
Average	18	30.00%	22	36.70%
High	0	0.00%	38	63.30%
Total	60	100%	60	100%

**Table 3:** Mean and standard deviation of Pre-test and Post-test level of self-esteem among adolescents

**Group**	**Mean**	**Mean Difference**	**Standard Deviation**	**% of Meanscore**	**'t'value**	**'p'-value**
Pre-Test	11.33	9.83	1.2	37.70%	T=33.4TV=3.5	P=0.001***
Post-Test	21.16		1.9	73%		

**Table 4:** Association between the levels of self-esteem among adolescents with their selected socio-demographic variables

**Sr**	**Variables**	**Categories**	**Level of self-esteem gain score**				**Total**
			**Below**		**Above**		
			**Average (11.38)**		**Average (>11.38)**		
			F	%	F	%	
1.	Age in years	13-15 Years	13	21.68%	3	5%	16
		15-17 Years	21	35%	8	13.30%	29
		17-19 Years	8	13%	7	25%	15
2.	School Performance	Good	1	1.66%	8	13.33%	9
		Average	24	40%	8	13.33%	32
		Poor	17	28.33%	2	3.33%	19
3.	Religion	Hindu	31	51.66%	9	15%	40
		Muslim	1	1.66%	0	0%	1
		Christian	7	11.66%	8	11.66%	15
		other	3	5%	1	1.66%	4
4.	Gender	Girl	15	25%	8	11.66%	23
		Boy	26	43.33%	11	18.33%	37
5.	Types of Family	Nuclear Family	24	40%	11	18.33%	35
		Joint Family	18	30%	7	11.66%	25
6.		<Rs.15000	6	10%	2	3.33%	8
		Rs.15001-25000	11	18.33%	6	10%	17
		Rs.25001-50000	18	30%	7	11.60%	25
	Monthly Income	>50000	7	11.66%	3	5%	10
7.	Area of residence	Urban	25	41.66%	10	16.66%	35
		Rural	17	28.33%	8	13.33%	25
8.	Co-Curricular Activity	Sports	28	83.33%	5	8.33%	30
		Yoga	8	13.33%	10	16.66%	18
		Other	9	15%	3	5%	12
